# Microbicides & their implications in HIV prevention

**Published:** 2010-12

**Authors:** Salim S. Abdool Karim, Cheryl Baxter

**Affiliations:** * Centre for the AIDS Program of Research in South Africa - CAPRISA, University of KwaZulu-Natal, South Africa; ** Department of Epidemiology Columbia University, New York, USA

Nearly half of the 33.4 million people living with HIV/AIDS worldwide are women^1^. In sub-Saharan Africa, women account for 59 per cent of all infected adults. Young women are especially vulnerable. Worldwide, 60 per cent of the 15 to 24 yr olds with HIV are women and between 70 and 90 per cent of all HIV infections among women are due to heterosexual intercourse. In sub-Saharan Africa, women aged 15 to 24 yr with HIV represent 76 per cent of the total cases in that age group, outnumbering their male peers by three to one^2^,^3^. In 2008, an estimated 4.7 million (3.8-5.5 million) people in Asia were living with HIV. India contributes approximately half of Asia’s HIV prevalence and in 2007 an estimated 39 per cent of all HIV infections in India were among women^1^.

In addition to biological factors^4^–^6^ that make women more vulnerable than men to acquiring HIV during sex, various sexual coupling patterns place young women at high risk, including partnering with older men who are more likely to be infected^7^, multiple concurrent relationships^8^, low marriage rates^9^, low consistent condom use rates^10^–^11^, and limited skills in negotiating safer sex practices. Gender-based violence increases vulnerability^12^, and poverty increases reliance on transactional sex for survival^13^. Women are often unable to convince their male partners, especially husbands and regular partners, to use condoms. Despite the greater vulnerability of women, they have very few options to reduce the transmission and acquisition of HIV. New technologies to prevent the sexual transmission of HIV in women are urgently needed.

## A brief history of microbicides

An HIV prevention strategy that women can initiate or control in the form of a microbicide was first proposed almost two decades ago^14^. Since then several candidate microbides have entered effectiveness trials to assess their impact on the prevention of HIV infection from products that disrupt cell membranes (surfactants such as nonoxynol-9 and C31G), or prevent attachment to target cells in the vagina (polyanions), to products (e.g., BufferGel) which maintain low vaginal *p*H in the presence of ejaculate. Most of the candidate microbicides that have been tested in late stage prevention trials have not shown protection against HIV infection^15^–^20^, and some products were even potentially harmful^15^,^21^,^22^. It is noteworthy that surfactants, polyanions and acid buffers are less specific to HIV than antiretrovirals^23^. Further, the antiviral activity of previous microbicide candidates was in the vaginal lumen compared to antiretroviral drugs which act against HIV replication inside vaginal CD4 positive target cells.

Currently, research on microbicides is focused on assessing potential antiretroviral agents for their ability to prevent HIV infection. The candidate microbicide in the most advanced stages of effectiveness testing is tenofovir gel. Tenofovir, an adenosine nucleotide analogue with potent activity against retroviruses^24^, was initially developed and tested as a prophylactic in monkeys and was subsequently formulated for oral use as tenofovir disoproxil fumarate (Viread^®^), which is now widely used for HIV treatment. Tenofovir’s efficacy in suppressing viral replication, favourable safety profile and long half-life^25^, made it an ideal choice as the first antiretroviral drug to be formulated as a microbicide gel. *In vitro* and *in vivo* assessments of the 1 per cent concentration of tenofovir in a gel formulation have demonstrated its potential as a microbicide^25^. Tenofovir has shown efficacy against viral challenge in animal models when administered as pre- or post-exposure prophylaxis^26^,^27^. In monkey challenge studies, tenofovir gel has shown protection with intermittent dosing and with a single pre-exposure dose^28^. In early stage clinical trials, tenofovir gel was well tolerated in both HIV negative and HIV positive women^29^, with both daily and coitally-related use of the gel being found to be acceptable and safe^30^.

## Results of the CAPRISA 004 tenofovir gel trial

The CAPRISA 004 trial, the first trial to assess the effectiveness of a 1 per cent vaginal gel formulation of tenofovir as a potential microbicide, was a doubleblind, randomized placebo-controlled trial which included 889 rural and urban South African women (ages 18 to 40 yr) who were sexually active and HIV-negative. A total of 445 randomly assigned women received vaginal applicators containing a 1 per cent concentration of tenofovir gel, and 444 received applicators filled with a placebo gel that looks identical to the study gel but does not contain tenofovir. Study participants were counselled to apply no more than two gel doses in 24 hours; the first dose within 12 hours before sex and second dose as soon as possible within 12 hours after sex (BAT24 dosing regimen).

The CAPRISA 004 trial recently provided the first evidence that tenofovir gel used before and after sex can prevent HIV infection in women^31^ contrary to past predictions^32^. HIV incidence in the tenofovir gel arm was 5.6 per 100 women-years (wy), compared to 9.1 per 100 wy in the placebo gel arm [Incidence Rate Ratio (IRR)=0.61; *P*=0.017]. In high adherers (gel adherence >80%), HIV incidence was 54 per cent lower (*P*=0.025) in the tenofovir gel arm. In intermediate adherers (gel adherence 50-80%) and low adherers (gel adherence < 50%) the HIV incidence reduction was 38 and 28 per cent respectively. Therefore, tenofovir gel reduced HIV acquisition by an estimated 39 per cent overall, and by 54 per cent in women with high gel adherence^31^. Tenofovir gel was also shown to be 51 per cent effective (*P*=0.003) in preventing Herpes simplex type 2 virus^33^.

## Unique features of the CAPRISA 004 trial

The CAPRISA 004 trial was unique in several ways. Firstly, it was the first effectiveness study of an antiretroviral drug formulated as a vaginal gel for prevention of HIV. Secondly, it was also the first microbicide trial where the consortium of partners was led by a developing country institution-the Centre for the AIDS Programme of Research in South Africa (CAPRISA), based at the University of KwaZulu-Natal in Durban, South Africa. Thirdly, it was the first microbicide trial that was co-funded by both the US government (through the United States Agency for International Development) and a developing country government agency, namely the Technology Innovation Agency (TIA) - a biotechnology agency of the South African government’s Department of Science and Technology. CAPRISA 004 was the first microbicide trial where a royalty-free voluntary license for local manufacture and distribution had been secured up front.

## Next steps

The promising findings of the CAPRISA 004 study are only a first step in determining if tenofovir gel is effective in preventing HIV; additional studies are urgently needed to confirm and extend the findings of the study. A critical step toward licensure and access of tenofovir gel is to conduct additional placebocontrolled studies that will help to confirm tenofovir gel’s level of effectiveness. The Microbicide Trials Network’s VOICE study is a crucial ongoing study testing daily use of tenofovir gel, as well as tenofovir tablets, in another HIV prevention approach called pre-exposure prophylaxis (PrEP). VOICE is expected to report results in 2013. VOICE will provide critically important information about whether daily tenofovir gel is safe and reduces the risk of HIV infection, and whether the level of protection is similar to or different from tenofovir tablets.

In addition to the ongoing VOICE study, two other placebo-controlled effectiveness studies are being proposed, each of which could provide valuable data needed for regulatory approval. The Follow-on African Consortium for Tenofovir Studies (FACTS) is planning a two-arm placebo-controlled safety and effectiveness study testing tenofovir gel using the same BAT24 coitally-related dosing regimen as used in the CAPRISA 004 trial in 16 - 30 yr olds. The Microbicides Development Programme (MDP) is planning a 4 arm placebo-controlled trial testing the BAT24 dosing regimen and a single pre-sex dose of tenofovir gel.

CAPRISA is planning implementation studies in the communities where the CAPRISA 004 trial took place. Trial participants and other women from the study communities will be eligible to enroll in these studies, which aim to address critical implementation questions about how best tenofovir gel could be incorporated into current health systems and made accessible to women who would benefit most from this product while also providing a mechanism for ongoing access to the tenofovir gel in these communities.

If proven effective, tenofovir gel could be an important prevention approach for many women who cannot rely on abstinence or on their male partners to use condoms. According to mathematical modeling, tenofovir gel has the potential to alter the course of the HIV epidemic. It is estimated that over the next two decades, this gel could prevent 1.3 million new HIV infections and over 800,000 deaths in South Africa alone^34^. Implemented on a broader scale, tenofovir gel could save millions of lives over time thereby helping to ease the global burden of providing treatment and care. The onus is on each of us to ensure that we do not miss this opportunity to translate a clinical trial result into public health impact.
